# Effect of supplementation of lysine producing microbes vis-a-vis source and level of dietary protein on performance and egg quality characteristics of post-peak layers

**DOI:** 10.14202/vetworld.2015.453-460

**Published:** 2015-04-09

**Authors:** G. U. Manju, B. S. V. Reddy, Gideon Gloridoss, T. M. Prabhu, K. S. Giridhar, N. Suma

**Affiliations:** 1 Department of Animal Nutrition, Livestock Research and Information Center, Karnataka Veterinary and Fisheries Science University, Nagamangala, Mandya, Karnataka, India; 2 Department of Animal Nutrition Veterinary College, Karnataka Veterinary and Fisheries Science University Hebbal, Bangalore, Karnataka, India; 3 Department of Animal Nutrition, Veterinary College, Karnataka Veterinary and Fisheries Science University, Hebbal, Bangalore, India; 4 Department of Animal Nutrition, Veterinary College, Karnataka Veterinary and Fisheries Science University, Shivamogga, Karnataka, India; 5 Department of Animal Nutrition, Veterinary College, Karnataka Veterinary and Fisheries Science University, Hassan, Karnataka, India

**Keywords:** egg characteristics, lysine, lysine producing microbes, post peak layers, performance

## Abstract

**Aim:**

The aim was to study the effect of supplementation of lysine producing microbes (LPM) as an *in vivo* source of lysine on performance and egg quality characters of post-peak layers.

**Materials and Methods:**

BIS (1992) specified diets (except crude protein [CP] and lysine) were prepared using either soybean meal (SBM) or groundnut extractions (GNE) or sunflower extractions (SFE) with 16 and 15% CP resulting in six control diets. Further, each control diet was fortified with either synthetic lysine or LPM to meet BIS (1992) specified lysine requirement resulting in the set of 12 test diets. Each of the eighteen diets was offered to quadruplets groups of 4 post-peak (52 weeks) commercial laying hens in each. The trial lasted for 119 days.

**Result:**

The results revealed that the feed consumption and body weight changes and Roche yolk color and yolk index were significantly (p ≤ 0.05) different among different treatments. However, egg production, feed efficiency, egg weight, egg shape index, Haugh unit score, albumen index and shell thickness, and net returns remained non-significant (p ≤ 0.05) among different treatments. Among main factors, protein level (16% and 15% CP) made a significant (p ≤ 0.05) difference in egg production (79.6 and 75.1%) and feed efficiency (2.64 and 2.81 kg feed/kg egg mass, respectively). Among protein source GNE- and SFE-based diet fed groups showed significantly (p < 0.0%) higher feed consumption and body weight gain than SBM based diets fed birds. Yolk color (7.0, 7.3 and 7.3, respectively) and yolk index (0.40, 0.38 and 0.43, respectively) were significantly (p ≤ 0.05) different from the protein sources. CP level and Protein source interaction effects showed significant differences in albumen index and Haugh unit score.

**Conclusion:**

Optimum level of protein (16% CP) and GNE as a source of protein tended to be superior in increasing the performance and egg characteristics of post-peak layers and supplementation of lysine in either synthetic or LPM form found to be beneficial in optimizing their performance.

## Introduction

The profit in the poultry industry can be maximized by decreasing the feed cost which accounts 75% of the total cost of egg production. Several commercial guidelines for laying hens [1,2] were recommended for crude protein (CP) levels, which vary from 17.4% to 18.2% (19.1-20.0 g of CP/d) per hen. Which appeared to higher than the recommendation of many recent reports [3-6] higher levels of protein/amino acids in diet will increase nitrogen excretion, ammonia emission, and taxing the ecosystem by contaminating surface water bodies [7] also often result in higher feed cost. Blair *et al.*,[8] obtained optimum layer performance when they were fed on low-protein diet (13.5%), which was properly supplemented with essential amino acids compared to layers fed 17% CP diet. Several studies have been undertaken on alternatives that might reduce CP level and cost of feed supplements with better economic results [9].

Supplementation of the low protein diets with crystalline amino acid is becoming relevant in feed formulation to minimize the nitrogen excretion and cost of production [10]. Lysine being the second most essential amino acid in the diet of poultry playing important vital function in egg production in layers and cereals used in feed composition contain low level of lysine [11], hence it is normally supplemented in the diet in the form of synthetic lysine (a product of microbial fermentation). The supplementation of lysine in a low protein diet has been found to increase egg mass, egg weight, and feed conversion in a sunflower seed cake based diet [8,12,13] reported that performance of layers fed control diet (16% CP) were comparable to those on the low-protein diet (14%) supplemented with essential amino acids. Supplementation of lysine may be in the form of synthetic lysine or any probiotic preparation, which can specifically synthesize adequate amount of lysine in the gut of the bird by utilizing the prevalent nitrogen. In addition to this, probiotics like *Lactobacillus* species [14] in layer diets did positively influence hen day egg production, feed conversion ratio (FCR), egg weight, and albumen quality, while decreasing serum cholesterol level. Dilworth and Day [15] reported that the supplementation of certain probiotics can specifically synthesize adequate amount of lysine in the gut of the bird by utilizing the prevalent nitrogen. Probiotics such as *Lactobacillus* species in layer diets did positively influence hen day egg production, feed efficiency, and egg weight [14].

Keeping in view the aforesaid facts, a comparative study was designed to put on record the effect of lysine producing microbes, a mixed culture of about ten microbes which can produce lysine in the gut of the birds compared with that of synthetic lysine in post-peak commercial layers using soya or groundnut extraction or sunflower extraction protein source based diets at two levels of dietary protein.

## Materials and Methods

### Ethical approval

This research work was carried out as per the guidelines in force at the time of carrying out the experiment as well as in accordance with the International Ethics Committee guidelines to minimize pain or discomfort of the birds. The study was approved by the Institutional Ethics Committee.

### Experimental design

A set of 18 experimental diets (Table-1) were formulated in a factorial design with two protein levels (16 and 15% CP), three protein sources (soybean meal [SBM], groundnut extractions [GNE], and sunflower extractions [SFE]) and with three lysine sources (no added lysine, synthetic lysine, and lysine producing microbes [LPM]). The LPM used in this study has been claimed to contain live microbial cultures of *Bacillus subtilis, Trichoderma reessi, Corynebacterium glutamicum, Cellulomonas uda, Alcaligenes faecalis, Conidiobolus coronatus, Penicilium roquefortii, Aspergillus oryzae, Aspergillus niger, Sachharomyces cereviseae* with a total viable count of 9000 million/g producing 1.238 g/day of L-Lysine *in situ* in bird in a 24 h period when fed at the rate of 1g LPM/bird. Such formulated diets with synthetic lysine or LPM irrespective of protein source provided 726 mg (16% CP) and 626 mg of lysine/kg (15% CP) while SBM, GNE, and SFE based diets without added lysine provided 706, 591, 593 (16% CP) and 646, 553 and 546 mg lysine/kg (15% CP), respectively. The ingredient and nutrient composition of the experimental diets are depicted in Table-2. The samples of experimental diets compounded on 5 occasions were analyzed for proximate principles as per AOAC [16]. 288 BV-300 commercial layers of 51-weeks age having uniform weight of 1.2-1.3 kg were selected and distributed randomly into 18 treatments comprising 16 birds (4 replicates with 4 birds in each replicate) in each treatment group. The birds were maintained under standard management conditions. The experimental period lasted for 119 days, which was conveniently divided into three 28-day interval periods.

**Table-1:** Ingredient composition of experimental layer diets.

Ingredients, kg	16% CP									15% CP								
	
	SBM			GNE			SFE			SBM			GNE			SFE		
					
	T_1_	T_2_	T_3_	T_4_	T_5_	T_6_	T_7_	T_8_	T_9_	T_10_	T_11_	T_12_	T_13_	T_14_	T_15_	T_16_	T_17_	T_18_
Maize	438	438	438	460	460	460	495	495	495	441	441	441	460	460	460	496	496	496
De oiled rice bran	271	271	271	231	231	231	64	64	64	300	300	300	266	266	266	110	110	110
SBM	170	170	170	0	0	0	50	50	50	138	138	138	0	0	0	36.5	36.5	36.5
GNE	0	0	0	188	188	188	50	50	50	0	0	0	153	153	153	36.5	36.5	36.5
SFE	0	0	0	0	0	0	220	220	220	0	0	0	0	0	0	200	200	200
Dicalcium phosphate	12.5	12.5	12.5	12	12	12	13.5	13.5	13.5	13	13	13	12	12	12	13.5	13.5	13.5
Calcite powder	29.5	29.5	29.5	30	30	30	28.5	28.5	28.5	29	29	29	30	30	30	28.5	28.5	28.5
Shell grit	75	75	75	75	75	75	75	75	75	75	75	75	75	75	75	75	75	75
Salt	3	3	3	3	3	3	3	3	3	3	3	3	3	3	3	3	3	3
Trace minerals	1	1	1	1	1	1	1	1	1	1	1	1	1	1	1	1	1	1
Total (kg)	1000	1000	1000	1000	1000	1000	1000	1000	1000	1000	1000	1000	1000	1000	1000	1000	1000	1000
Additives kg/ton																		
Toxin binder	0.75	0.75	0.75	0.75	0.75	0.75	0.75	0.75	0.75	0.75	0.75	0.75	0.75	0.75	0.75	0.75	0.75	0.75
Choline chloride	0.50	0.50	0.50	0.50	0.50	0.50	0.50	0.50	0.50	0.50	0.50	0.50	0.50	0.50	0.50	0.50	0.50	0.50
DL-Methionine	0.60	0.60	0.60	0.60	0.60	0.60	0.60	0.60	0.60	0.60	0.60	0.60	0.60	0.60	0.60	0.60	0.60	0.60
Lysine	0.00	0.20	0.00	0.00	1.35	0.00	0.00	1.33	0.00	0.00	0.20	0.00	0.00	1.13	0.00	0.00	120	0.00
LPM	0.00	0.00	0.31	0.00	0.00	2.10	0.00	0.00	2.07	0.00	0.00	0.31	0.00	0.00	1.76	0.00	0.00	1.87

LPM=Lysine producing microbes, SBM=Soya bean meal, SFE=Sunflower extractions, GNE=Groundnut extractions, CP=Crude protein

**Table-2:** Proximate composition of experimental diets^1^ (% on DM basis).

Protein level	Protein source	Lysine source	Lysine^2^	Trade No.	Dry matter	Organic matter	Total ash	CP	Crude fiber	Ether extract	NFE
16% CP	SBM	Control	0.706	T_1_	90.5	81.1	18.9	15.4	7.2	2.6	55.9
		Syn. Lysine	0.726	T_2_	89.4	82.8	17.2	15.8	7.3	1.9	57.8
		LPM	0.726	T_3_	90.1	82.2	17.8	15.3	7.8	3.3	55.8
	GNE	Control	0.591	T_4_	89.7	79.8	20.2	15.3	7.1	2.9	54.5
		Syn. Lysine	0.726	T_5_	89.5	83.8	16.2	15.7	7.2	3.1	57.8
		LPM	0.726	T_6_	90.2	84.2	15.8	15.8	7.0	2.7	58.7
	SFE	Control	0.593	T_7_	91.5	83.1	16.9	15.2	7.8	2.0	58.1
		Syn. Lysine	0.726	T_8_	89.2	84.2	15.8	15.0	7.7	2.1	59.4
		LPM	0.726	T_9_	90.5	85.5	14.5	14.9	7.8	3.0	59.8
15% CP	SBM	Control	0.646	T_10_	89.4	81.6	18.4	14.4	7.2	3.4	56.6
		Syn. Lysine	0.666	T_11_	90.3	86.3	13.7	14.3	7.3	3.1	61.6
		LPM	0.666	T_12_	89.7	83.4	16.6	14.8	7.4	3.8	57.4
	GNE	Control	0.553	T_13_	90.1	85.7	14.3	14.2	7.2	3.6	60.7
		Syn. Lysine	0.666	T_14_	89.4	84.3	15.7	14.1	7.2	2.6	60.4
		LPM	0.666	T_15_	89.3	83.4	16.6	13.9	7.2	2.2	60.1
	SFE	Control	0.546	T_16_	90.7	82.9	17.1	14.4	7.8	2.7	58.0
		Syn. Lysine	0.666	T_17_	91.2	77.9	22.1	14.1	7.8	2.8	53.2
		LPM	0.666	T_18_	89.5	78.1	21.9	14.3	7.8	2.7	53.3

LPM=Lysine producing microbes, SBM=Soya bean meal, SFE=Sunflower extractions, GNE=Groundnut extractions, CP=Crude protein, NFE=Nitrogen-free extract,

1 Average values of compounded diets on five occasions,

2 Calculated values

### Performance evaluation

The daily required quantity of specified diet was collectively offered to each replicate of four birds in divided dose of about 50% in morning and remaining 50% in the afternoon hours. All the birds were weighed individually at the beginning of the experiment as well as at every 28 days interval to monitor the pattern of body weight changes if any due to dietary regimen. The average body weight in the replicate group was then calculated. Egg produced from each replicate were recorded and weighed twice (Tuesday and Friday) in a week of the experimental period. The feed efficiency was calculated based on the amount of feed consumed to produce one kg egg mass as per replicate. On the terminal day of every 28-day interval, all the eggs produced from different replicate groups were collected and were weighed individually during the experimental period of 84 days. Further, on the immediate next day, each egg was broken and the entire contents were carefully placed on a glass slab for egg quality study. Albumen index score was calculated as the ratio height and diameter of thick albumen (in mm) which were measured by Ames^®^ spherometer and by Vernier Caliper, respectively. Yolk index score, the ratio of height to diameter of yolk calculated in similar way. Egg shape index expressed as a relationship of length and breadth of the egg (in cm) obtained using Vernier Calipers. The height of albumin was recorded at two consistent places using Ames Haugh unit meter to obtain the Haugh unit score.


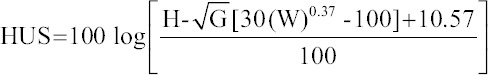


H: is the height of albumen (mm)

G: Gravitational force (981 cm/s; w weight of the egg)

The shell pieces devoid of shell membranes at a broad end, narrow end, and middle band were selected and their thickness was measured using a digital calipers. The average of all the three pieces was represented as shell thickness. The color of the yolk of every broken open egg was scored by matching (contrast) technique using Roche yolk color fan [17].

### Statistical analysis

The data generated during the experiment were statistical analyzed by completely randomized design as well as by factorial design according to the methods described by Snedecor and Cochran [18].

## Results

### Production characteristics

The performance of layers under different treatment is presented in Table-3. The average daily feed consumption ranged significantly (p ≤ 0.05) from 117.1 (16% CP, SBM, Syn. Lysine) to 127.5 g (15% CP, GNE, LPM). The cumulative average egg production varied from as low as 70.6 (15% CP, SBM, no lysine) to as high as 85.7% (16% CP, GNE, no lysine) however the differences were non-significant and no definitive trend was observed among different treatments. The feed efficiency, body weight gain, and net returns also remained non-significant among different treatments. Table-4 shows the influence of different main factors, 16 and 15% levels of protein showed significant (P ≤ 0.05) differences in egg production and feed efficiency. The egg production and feed efficiency was 79.6; 2.64 and 75.1; 2.82%, respectively, at 16 and 15% CP level in the diets. However, the difference in feed consumption, body weight change, and net returns were non-significant (p > 0.05).

**Table-3:** Performance of layers under different treatments.

Treatment description					Feed consumption* g/hen/day	Body weight gain, g/hen	Egg production, %	Feed efficiency, kg feed/kg egg mass	Net returns. (Rs/28 day)

Protein level	Protein source	Lysine source	Lysine %^1^	Trade No.					
16% CP	SBM	Control	0.706	T_1_	121.3^abc^±1.2	33.1±12.1	77.5±1.7	2.68±0.2	5.5±±1.1
		Syn. Lysine	0.726	T_2_	117.1^a^±0.9	17.4±4.4	85.0±1.8	2.32±0.1	5.8±1.1
		LPM	0.726	T_3_	117.3^a^±1.6	21.7±17.1	75.9±3.9	2.78±0.1	3.7±1.1
	GNE	Control	0.591	T_4_	126.8^c^±1.3	33.8±3.3	85.7±1.3	2.51±0.1	2.9±0.8
		Syn. Lysine	0.726	T_5_	124.3^bc^±1.4	40.4±12.5	77.5±2.4	2.71±0.2	4.1±0.9
		LPM	0.726	T_6_	125.2^bc^±1.1	45.6±22.8	84.3±1.8	2.53±0.1	6.2±1.6
	SFE	Control	0.593	T_7_	124.0^bc^±1.1	63.5±16.9	74.1±2.2	2.85±0.2	4.0±1.7
		Syn. Lysine	0.726	T_8_	119.1^ab^±3.2	72.0±20.0	77.7±3.2	2.65±0.3	4.4±1.9
		LPM	0.726	T_9_	127.0^c^±2.8	58.7±23.0	77.7±3.2	2.71±0.2	3.9±1.9
15% CP	SBM	Control	0.646	T_10_	118.2^a^±1.9	28.7±17.5	70.6±2.9	2.91±0.2	3.3±2.0
		Syn. Lysine	0.666	T_11_	117.6^a^±1.7	45.2±19.3	74.9±3.4	2.73±0.2	5.3±1.0
		LPM	0.666	T_12_	121.4^abc^±1.4	28.3±21.5	81.9±2.7	2.53±0.2	5.5±1.3
	GNE	Control	0.553	T_13_	125.8^c^±1.6	50.5±20.8	72.6±5.8	2.83±0.3	3.4±1.0
		Syn. Lysine	0.666	T_14_	124.9^bc^±1.3	48.2±15.8	71.1±2.1	3.06±0.2	4.2±0.9
		LPM	0.666	T_15_	127.5^c^±1.3	34.5±17.4	77.4±2.5	2.84±0.1	3.8±0.7
	SFE	Control	0.546	T_16_	125.0^bc^±1.3	35.1±19.8	78.4±2.7	2.79±0.2	3.0±1.5
		Syn. Lysine	0.666	T_17_	125.4^c^±1.6	46.3±2.8	75.7±2.3	2.86±0.1	3.8±1.3
		LPM	0.666	T_18_	124.1^bc^±1.7	35.4±4.8	72.7±2.2	2.84±0.1	2.3±1.3
			SEM		1.40	41.20	4.60	0.40	2.3

LPM=Lysine producing microbes, SBM=Soya bean meal, SFE=Sunflower extractions, GNE=Groundnut extractions, CP=Crude protein,

* Means with common superscripts are statistically similar (p<0.05),

1 Calculated lysine values, SEM=Standard error of the mean

Among protein sources, the SBM based diet group of layers recorded significantly (p < 0.05) lower daily feed consumption (118.9 g) as against those of GNE (125.8 g) and SFE (124.1 g) groups. Weight gain was significantly higher in SFE (51.8) with lowest being SBM group (31.1). Egg production was 78.2 per cent in GNE groups while that of SBM (77.7%) and SFE (76.1%) groups which were statistically similar (p ≤ 0.05). Sources of lysine did not show any significant difference in production parameters. Various interaction effects (Table-4) *viz*., protein level × protein source, protein levels × supplemented lysine source and protein source × lysine source interaction also remained statistically non-significant (p > 0.05) in influencing the feed consumption, body weight gain, egg production, and net returns.

**Table-4:** Performance of layers as influenced by various main factors and interaction effects in the diet.

Main factors	Feed consumption g/hen/day	Body weight gain, g/hen	Egg production, %	Feed efficiency, kg feed/kg egg mass	Net returns. (Rs/28 days)
Protein level					
16% CP	122.6±0.5	42.9±23.6	79.6^b^±6.5	2.64^a^±0.3	4.5±1.6
15% CP	123.4±0.1	39.1±3.14	75.1^a^±5.9	2.82^b^±0.1	3.8±2.0
Protein source					
SBM	118.9^a^±0.3	31.1^a^±31.1	77.7±4.3	2.66±0.2	4.8±1.4
GNE	125.8^b^±0.7	40.2^b^±42.3	78.2±8.5	2.75±0.4	4.1±1.2
SFE	124.1^b^±0.3	51.8^c^±51.8	76.1±6.4	2.78±0.1	3.6±2.3
Lysine source					
Control	123.6±1.3	38.8±40.9	76.6±7.3	2.76±0.5	3.7±2.7
Syn lysine	121.5±0.2	44.9±44.9	77.0±6.8	2.72±0.4	4.6±2.1
LPM	123.8±0.8	39.4±±39.4	78.4±8.2	2.71±0.3	4.2±3.4
SEM	1.40	41.20	4.60	0.40	2.30
Significance					
CP level	NS	NS	p<0.05	p<0.05	NS
Protein source	p<0.05	p<0.05	NS	NS	NS
Lysine source	NS	NS	NS	NS	NS
CP level × protein source interaction	NS	NS	NS	NS	NS
CP level × lysine source interaction	NS	NS	NS	NS	NS
Protein source × lysine source interaction	NS	NS	NS	NS	NS

NS=Non-significant;

a,b Mean values with different superscripts with in a column differ significantly, LPM=Lysine producing microbes, SBM=Soya bean meal, SFE=Sunflower extractions, GNE=Groundnut extractions, CP=Crude protein

### Egg quality characters

Level of protein, source of protein and lysine sources were non-significant in influencing egg weight, shape index, albumen index, Haugh unit score and shell thickness (Table-5). However, the yolk color was significantly more in both GNE and SFE based diet and yolk index was highest in GNE based diets. Table-5 illustrate the interactions of the protein level x protein source, the SFE based 15% CP group has shown significantly (p ≤ 0.05) lower Haugh unit score (64.7) when compared to SFE based 16% group (68.1). Various treatments (Table-6), main factors and interaction effects showed non-significant (p > 0.05) influence on the average egg weight, egg shape index, albumen index, Haugh unit score, yolk color, egg shell thickness, and yolk index except CP level protein source interaction for albumen index, yolk index, and Haugh unit score.

**Table-5:** Egg quality characteristics of layers.

Treatment description					Egg weight	Egg shape index	Albumen index	Haugh unit score	Yolk color*	Yolk index*	Shell thickness

Protein level	Protein source	Lysine source	Lysine %^1^	Trade No.							
16% CP	SBM	Control	0.706	T_1_	56.9±0.8	70.1±4.4	0.048±0.05	64.2±1.7	6.9^ab^±1.72	0.36^ab^±0.03	0.35±0.00
		Syn. Lysine	0.726	T_2_	58.4±1.3	75.6±0.5	0.055±0.06	66.5±1.5	7.2^abcde^±1.79	0.40^b^±0.01	0.35±0.01
		LPM	0.726	T_3_	57.2±1.0	75.4±0.6	0.056±0.05	66.0±1.4	7.0^abc^±1.66	0.40^ab^±0.41	0.34±0.23
	GNE	Control	0.591	T_4_	57.2±1.0	70.4±4.5	0.060±0.06	65.2±2.7	7.2^abcde^±1.79	0.33^a^±0.04	0.35±0.01
		Syn. Lysine	0.726	T_5_	58.5±1.1	75.9±0.4	0.063±0.05	67.9±1.9	7.5^e^±1.86	0.35^ab^±0.02	0.35±0.01
		LPM	0.726	T_6_	57.2±0.8	76.6±0.5	0.056±0.02	66.1±1.7	7.3^cde^±1.82	0.37^ab^±0.02	0.35±0.01
	SFE	Control	0.593	T_7_	57.7±0.9	76.7±0.7	0.059±0.01	67.8±1.6	7.3^cde^±1.82	0.41^b^±0.08	0.36±0.01
		Syn. Lysine	0.726	T_8_	57.4±0.9	73.4±2.2	0.057±0.05	68.0±1.3	7.2^bcde^±1.87	0.37^ab^±0.03	0.35±0.01
		LPM	0.726	T_9_	57.4±0.9	76.5±0.4	0.056±0.05	68.2±1.4	7.1^abcd^±1.80	0.42^bc^±0.01	0.34±0.05
15% CP	SBM	Control	0.646	T_10_	57.9±0.8	76.6±1.7	0.056±0.04	67.1±4.5	7.1^abcd^±1.81	0.41^b^±0.02	0.36±0.01
		Syn. Lysine	0.666	T_11_	57.7±1.0	76.2±0.7	0.061±0.03	68.2±1.7	7.2^abcde^±1.81	0.39^ab^±0.02	0.35±0.00
		LPM	0.666	T_12_	57.8±0.7	76.3±0.3	0.057±0.07	67.9±1.3	6.8^a^±1.71	0.40^b^±0.02	0.35±0.01
	GNE	Control	0.553	T_13_	58.2±0.9	76.0±0.9	0.059±0.09	68.0±1.3	7.1^abcde^±1.81	0.41^b^±0.01	0.35±0.01
		Syn. lysine	0.666	T_14_	58.0±0.8	75.2±0.3	0.056±0.04	67.7±1.0	7.3^cde^±1.84	0.37^ab^±0.02	0.35±0.01
		LPM	0.666	T_15_	57.5±0.8	77.0±0.7	0.055±0.09	67.1±1.1	7.4^de^±1.86	0.42^b^±0.02	0.35±0.01
	SFE	Control	0.546	T_16_	57.8±0.8	76.4±0.5	0.048±0.04	62.2±1.2	7.3^cde^±1.83	0.41^b^±0.01	0.35±0.01
		Syn. lysine	0.666	T_17_	57.5±0.9	75.6±0.3	0.055±0.07	66.1±1.2	7.5^e^±1.86	0.50^c^±0.01	0.35±0.01
		LPM	0.666	T_18_	57.7±1.0	75.0±0.4	0.054±0.08	65.8±1.6	7.4^de^±1.84	0.43^bc^±0.01	0.35±0.01
			SEM		1.5	0.50	0.07	1.90	0.24	0.003	0.005

LPM=Lysine producing microbes, SBM=Soya bean meal, SFE=Sunflower extractions, GNE=Groundnut extractions, CP=Crude protein, Values with different superscript are significantly different at P<0.05

**Table-6:** Egg quality characteristics of layers as influenced by various main factors and interaction effects in the diet.

Main factors	Egg weight	Egg shape index	Albumen index	Haugh unit score	Yolk color	Yolk index	Shell thickness
Protein level							
16% CP	57.6±0.2	74.6±0.9	0.057±0.08	66.6±0.6	7.2±0.5	0.38±0.06	0.37±0.05
15% CP	57.8±0.2	76.1±0.4	0.056±0.06	66.7±0.9	7.2±0.4	0.42±0.07	0.35±0.04
Protein source							
SBM	57.7±0.3	75.1±0.4	0.055±0.04	66.7±1.8	7.0^a^±0.6	0.40^a^±0.05	0.33±0.06
GNE	57.8±0.4	75.3±0.2	0.058±0.04	67.0±2.0	7.3^b^±0.6	0.38^a^±0.04	0.31±0.01
SFE	57.6±0.2	75.6±0.1	0.055±0.02	66.4±1.4	7.3^b^±0.4	0.43^b^±0.01	0.35±0.02
Lysine source							
Control	57.7±0.6	74.5±0.2	0.055±0.04	66.7±1.8	7.1±0.1	0.39±0.08	0.35±0.03
Synthetic lysine	58.0±0.3	75.4±0.7	0.058±0.04	67.0±2.0	7.3±0.5	0.40±0.09	0.35±0.08
LPM	57.5±0.7	76.2±0.5	0.055±0.02	66.4±1.4	7.2±0.4	0.41±0.07	0.31±0.09
Significance							
CP level	NS	NS	NS	NS	NS	NS	NS
Protein source	NS	NS	NS	NS	p<0.05	p<0.05	NS
Lysine source	NS	NS	NS	NS	NS	NS	NS
CP level × Protein source interaction	NS	NS	p<0.05	p<0.05	NS	p<0.05	NS
CP level × Lysine source interaction	NS	NS	NS	NS	NS	NS	NS
Protein source × lysine source interaction	NS	NS	NS	NS	NS	NS	NS

NS=Non-significant, LPM=Lysine producing microbes, SBM=Soya bean meal, SFE=Sunflower extractions, GNE=Groundnut extractions, CP=Crude protein

## Discussion

### Production characteristics

Among different treatment, the feed consumption ranged significantly with, GNE-based diet fed group had highest feed consumption followed by that of SFE and SBM groups. Cumulative average egg production, feed efficiency, body weight gain, and net returns remained non-significant among different treatments. However, SFE-based groups showed numerically higher body weight gains (72 g) when compared to SBM based diet fed groups (17.4 g) which may be due to, relatively lower production (due to lower availability of lysine) in SFE group compared to soya groups leading to more body fat accumulation. Level of protein in the diet significantly affected the egg production and feed efficiency this is in contrary to Chaiyapoom *et al*. [19] were he reported non-significant difference in production and feed intake at 15% and 16% level of protein and Ji *et al*. [20] also reported that egg production, daily egg mass, feed intake, and FCR were not affected in the low-protein groups. Contrarily, Bustany and Elwinger [21] and Parla *et al*.[22] reported lower feed intake in low protein diets with lysine levels ranging between 0.46 and 0.66%. Among the protein sources, the SBM based diet group of layers recorded significantly lower feed consumption, which was in contrary to Rose *et al*. [23] who in their study replaced lysine rich SBM diet with lysine deficit SFE diets which failed to show any significant difference in the performance of layers. Though GNE had higher lysine: Arginine antagonism [24], yet in the present study, a better egg production with GNE protein source appears to be due to better provision of lysine either from synthetic or LPM source [25]. The egg production, feed efficiency or net returns remained similar among groups fed SBM or GNE or SFE protein based diets in the present study. Regarding lysine source, no significant differences were observed in feed consumption, egg production, body weight, feed efficiency or net returns in birds fed control, synthetic lysine or LPM supplemented diets. Similar findings with supplemental lysine levels of 0.85, 0.98 and 1.18% in the diets of post-peak layers was observed by Vogt and Krieg [26] and Prochaska *et al*. [27]. Kurtoglu *et al*. [28], Panda *et al*. [29], and Bonekamp *et al*. [30] observed non-significant differences in performance of layers with supplementation of commercial probiotic preparation. Control and LPM groups showed numerically higher feed consumption than synthetic lysine group. Such a trend is obvious since birds tend to consume more feed in the lysine deficiency [31] or the marginal deficiency of any specific amino acids [32] and in LPM supplemented group due to gut stabilization feed consumption in more [14]. Yalcin *et al*. [32] reported significant improvement (4.65%) in the egg production with a commercial probiotic preparation fed @ 0.75 kg/ton. Interactions of level of protein, sources of protein and lysine source did not show any significant difference. In contrary, Sherman *et al*. [25] and Parla *et al*. [22] noted better performance of birds fed low protein diets supplemented with lysine. In present study, SFE based protein with or without supplementation showed higher gain in body weight as compensatory to low egg production in these groups. Karunjeewa *et al*. [12] also reported higher gain in SFE group with lysine supplementation. The higher egg weights in GNE group were in effect due to higher feed consumption in GNE based group when compared to other groups and that the supplemented synthetic lysine has supported good egg weights. Bustany and Elwinger [21] and Sohail *et al*. [33] also reported numerical decrease in the feed efficiency in lysine deficit low protein group.

### Egg characteristics

Numerically, the data indicated better shape index with supplementation of LPM in low protein groups. The main factors *viz*. protein level (16 and 15% CP), protein source (SBM, GNE, and SFE) and lysine source (no added lysine, synthetic lysine, and LPM) showed non-significant difference on egg weight, shape index, albumen index, Haugh unit score, and shell thickness this was also reported by Figueiredo *et al*. [34], who registered no individual effects of amino acids in the proportion of egg components.

Numerically, LPM supplementation was found to increase shape index, yolk color and yolk index. These results are similar to those of Sohail *et al*. [33] who reported non-significant increase in the shape index with supplementation of lysine. Balvi *et al*. [35] also reported similar results with supplementation of Protexin (commercial probiotic preparation). With regard to supplemented LPM, which showed statistical non-significance, the trend was similar to that of synthetic lysine [24,32]. Low protein group showed relatively higher shape index because of larger eggs in this group, this was in agreement with the results of Adeyemo *et al*. [36] findings. Shim *et al*. [37] also reported that egg quality characters were similar regardless of level of protein in the diet. The GNE based optimum protein group might be good enough to provide better digestible protein to support albumen index. With the protein level × lysine source combination also, the low protein group with no supplementation of lysine (0.054) had comparatively lower albumen index in relation to optimum protein with Synthetic lysine supplementation (0.058). Prochaska *et al*. [27] showed similar results with supplementation of lysine. With regards to the protein source × lysine source, the SBM based control group showed lower albumen index (0.052) when compared to GNE based control and synthetic lysine supplemented group’s (0.059). Various treatments and interaction remained non-significant in influencing Haugh unit score, similar findings were reported with commercial probiotic (Biocell) supplementation [32]. Optimum protein (16% CP) with synthetic lysine supplementation showed better score (67.5) when compared to non-supplemented optimum protein group (65.6) similar to the reports of Novak *et al*. [38] and Trindade *et al* [39]. GNE-based synthetic lysine supplemented group showed better score (67.8) compared to SFE based non-supplemented group (65.1) and further that the influence of lysine supplementation did exert a positive influence. The dietary treatment with synthetic lysine has shown significant (p < 0.05) effect in improving the yolk color. The levels of protein showed non-significant difference, while protein sources, GNE and SFE significantly (p ≤ 0.05) increased the yolk color compared to SBM. Sohail *et al*. [33], also found marginal improvement in the yolk color but statistically it was non-significant. However, various interactions were tested statistically non-significant in influencing the yolk color. There was a significant (p < 0.05) variation in yolk index between different treatments. Among protein sources, birds fed SFE protein sourced diets showed significantly (p < 0.05) highest yolk index compared to GNE and SBM based diets fed groups. Supplementation of lysine in either forms was found to improve the yolk index, however, the differences were non-significant (p > 0.05). Among protein levels, low protein group (15% CP) showed higher index compared to optimum protein group (16% CP). It may be because of relatively lower production in low protein group leading to increased yolk size. Among the interaction effects, the SFE based low protein group showed significantly (p ≤ 0.05) higher yolk index (0.45) while GNE based optimum protein group was lowest (0.36). All the three levels of lysine in low protein group were of the same order in influencing the yolk index (0.42), but in optimum protein group with no supplementation it was the lowest index (0.36). Synthetic lysine supplemented SFE group showed higher index (0.44) compared to GNE based control and synthetic lysine groups (0.37). Various main factors and interactions showed statistical non-significance in influencing the average eggshell thickness. Among protein levels, optimum protein group showed better shell thickness compared to low protein group, which might have been due to an adequate supply of protein for shell matrix formation. SFE source protein showed thicker shell than GNE source because of lower egg production. LPM supplementation reduced the shell thickness.

It was concluded that the supplementation of lysine in the form of LPM was as good as synthetic lysine in optimizing the production performance of the birds; however, further studies are required in this direction to justify the above claim. Protein level was of greater significant in influencing the egg production and feed efficiency, indeed level of lysine to a some extent showed its importance but statistically it was not significant.

## Authors’ Contributions

This study was part of MVSc research program of GUM. BSVR designed the research program as mentor and GUM conducted the research work. GG and TMP worked as co – mentors for the MVSc program, provided technical guidance and also helped in preparing this manuscript. NS and KSG helped in carrying out research trial, laboratory analysis and in manuscript preparation All authors read and approved the final manuscript.
